# Are Our Dental Organizations Satisfactory?

**Published:** 1894-02

**Authors:** 


### Editorial.

### ARE OUR DENTAL ORGANIZATIONS SATISFACTORY?

The history of dental organizations throughout the world is, probably, very much the same, and it is presumed the record, if such exists, would show that the various bodies have originated through the aggregation of individuals without much or any attempt at classification. In some local societies, of latter years, a certain qualification is demanded, as a diploma from a recognized college or a medical degree. While this condition is an advance on previous loose methods, it does not seem to have helped very much the outcome, for, as a rule, results have by no means been equal to expectations.

Organizations in dentistry have from the beginning followed set lines. The custom adopted from time immemorial in the medical ranks has been the example followed, and that with a persistence that must be regarded as strange, when it is remembered that dentistry arose independently, and should have marked its own path and refused to be a mere copyist of the work of other men and other professions. It was original in most things, but in this respect it began exactly with the old methods, and has not made any marked advance since the first organization was completed.

Less than sixty years ago there was not a dental organization on this continent, the first formed being in New York in 1837. It is true that Dr. Hayden urged the idea as early as 1817, but there was no response to his efforts, and those who can recall the condition of dentistry as late as 1837 can only wonder that at that period any kind of an organization was possible. The dentists of that time surrounded themselves with a wall of exclusion, every man being practically at war with every other man in the practice of dentistry. It is, therefore, not surprising that the two original societies in New York died and left no history.

In 1840 the American Society of Dental Surgeons was organized with Dr. Hayden as President. From that period until the formation of the American Dental Association in 1855 there were but five associations organized,—the Virginia Society of Surgeon Dentists in 1842, the Mississipi Valley Association of Dental Surgeons in 1844, the Pennsylvania Association of Dental Surgeons, the Society of Dental Surgeons of the State of New York, and the successor of the American Society of Dental Surgeons, the American Dental Convention, organized in 1855. It would be difficult to number the local State and National Associations existing to-day, but except in minor matters they exhibit no improvement in their manner of work over those first called into being.

This would seem not to be on the line of progress. It is certainly true that dentistry has made marked advances in the period named. It has progressed very nearly to the standard of a learned profession, and its methods of legislation on scientific lines should have advanced with a proportionate rapidity.

It may truly be said that the work of building up the theoretical and practical details has been too exacting to permit of any originality in other directions. While this is acknowledged it will not much longer be a valid excuse. The imperfections now existing in society work will continue unless there be an effort made to change conditions and means of labor.

It is certainly true that while dental organizations have been of inestimable value in binding together and liberalizing the incongruous elements engaged in the practice of dentistry, and have proved most valuable incentives to scientific work, the real progress of dentistry is exclusively due to the work of individuals and largely independent of any society efforts.

There has been no attempt made, so far as we are aware, to crystallize this isolated scientific element into one body. It remains as it began, without organization and often without encouragement.

The evolution of societies throughout the United States presents a curious state of affairs. They may be classed as local, State, and national. In many sections of the country a practitioner must travel hundreds of miles to attend a meeting, and in other and more populous districts, as large cities, two and frequently three societies are found working with more or less success.

In the entire aggregation there is no system, if we exclude the State of New York. A few men get together and form a local society; a few more from different sections of a State organize a State society; a larger number from the nation develop a national organization. The local society may or may not require the degree for admission. The chords of fraternal labor are drawn closer here than elsewhere, hence we find that local societies practically perform all the real scientific work of the profession.

It is difficult at this date to determine why State organizations were ever deemed a necessity. It is presumed they originated under the idea that each State must be independent of every other State in the confederacy. It is certain that results do not warrant any great efforts for their continuance as a working part of the profession. While these profess to represent the associations of the various Commonwealths, they generally retain that odious clause of permanent membership in their constitutions similar to that of the National Association, than which there is nothing more fatal to representative character or scientific work.

When it is remembered that in eight of the States of the United States these irresponsible bodies have the nomination or appointment of the State Boards of Examiners, and in one State they appoint the majority of the Board, it seems as though the time had come for a reorganization in all directions, but especially should there be a change in the anomalous condition of State matters.

The attachment felt for the organizations supposed to represent the largest constituencies, the American Dental Association and the Southern Dental Association, make it a difficult task to effect any change there, but it must be evident to the members that the work performed by these Associations bears no relation to the expenditure of time and means for their maintenance. It will hardly be asserted that either of them have accomplished much scientific work. In the American there has been a notable and worthy effort the past three or four years in this direction, but every attempt there is hampered by an unwieldy organization, and in the end all results are attained by individual efforts.

The most convincing example of herculean effort without a corresponding equivalent in results has recently been given the world in the Columbian Dental Congress. It demonstrated, as never before, that it is impossible to bring masses of professional men together and in this way advance the standard of scientific knowledge. If dentistry is to develop beyond its present status, other means must be adopted, and we think the time is propitious for at least a discussion and comparison of views as to the best means to accomplish more with less use of dead material.

We think it must be conceded that the local societies, as now constituted, are, probably, the best form of organization, as they come closest to the individual. The State associations should be relegated to oblivion, as they can give no reasonable excuse for existence. The national organizations should be remodelled on strict scientific lines or be dropped altogether as having outlived their usefulness.

While the writer of this does not feel it his duty, even if he be competent, to suggest plans for reorganization, it may not be construed as out of place to offer a personal opinion.

If the individual is to be the unit in scientific investigation, the local society should be the basis of his support. These should be the feeders to a national organization, and there should be nothing between these two bodies.

Representation from the local to the national should be based solely on original work. Mere membership should have no weight in the selection. The national body would then be composed of a limited number, but all would be original thinkers. In this body a similar rule should be enforced compelling a member, during his continuance as a delegate, to present one or more truly original papers upon subjects worked up in the laboratory and not in the brain of the writer. The influence of such an organization cannot be computed. It would in a very brief period change the whole current of thought and challenge the respect of the world.

We do not propose to work out the details of such an organization. To many, doubtless, it will seem utopian, but in the view of the writer of this it must be the organization of the future, and it seems to him that it is the duty of all interested in the scientific well-being of our profession to work up towards it, and, in any event, leave time-worn roads for a more direct avenue to the Temple of Knowledge.

